# A Cold in the Head

**Published:** 1890-12

**Authors:** 


### A COLD IN THE HEAD.

BY DR. HANAFORD, IN THE "PHRENOLOGICAL JOURNAL."

A genuine cold is simply the closing of the pores of the surface, about seven millions in the human system, retaining a portion of the effete particles of the ever decaying body, more or less poisonous, this retention necessarily antagonizing good health ; the lungs—sustaining a very intimate relation to the skin—usually are more especially affected, performing some of the labors of the surface. It is usual, however, for the weaker parts of the system to take on diseased action, which is but another name for recuperative efforts, the efforts of nature, by apparently inimical means, pain producing, ordinarily—by which to remove a real difficulty, not always observed by the victim. Some of these efforts are regarded by the masses as "colds in the head," while it is probable that not more than one in ten of these supposed colds have any connection with the closing of the pores. Most, if not all, of the irritation in the nasal passages, the inflammation of the mucous surfaces, not only of the nasal passages, but of the throat, etc., with the sores about the nose and on the lips, usually regarded as "cold sores," have their origin in a deranged state of the stomach, the inner surface of this organ having a similar appearance. As a result of improper dietetic habits, taking food very difficult of digestion, too much of ordinary food, or at improper times, eating so rapidly that it is not half masticated, some have a continuous "head cold" and are unable to breathe with the mouth closed, thus inducing additional disease. The appropriate treatment of such supposed colds, etc., is the adoption of simple habits, careful dieting, making the grains and fruits more than usually prominent, eating flesh very sparingly—if at all—no pork, or any of the products of the filthy scavenger ! In these modern, progressive times there are so many excellent, nutritious, easily digested, delicate and palatable preparations from the grains, that none need select food of a doubtful quality, these preparations being sold for far less than the popular luxuries, although they contain much more nourishment. These preparations may, with great propriety, constitute a part of the morning meal or the dinner, while the majority of all communities would be benefited by using them as the only food taken at the latter meal. Such a course, with due care in all respects, would soon remove that "all gone feeling at the pit of the stomach," with other unpleasant sensations, when the "head colds"

would also disappear. I will add that these supposed colds have led many persons to take undue care of the head, in contrast with the feet, which demand a great deal more attention as the means of warding off such dreaded evils, such as wearing fur caps or close hats, and in audiences covering the head when there may be the slightest air stirring, etc., while those of reasonable intellect and a normal amount of hair should have sufficient brain activity to keep the head as warm as it should be, under ordinary circumstances.

